# Profiles of low complexity regions in Apicomplexa

**DOI:** 10.1186/s12862-016-0625-0

**Published:** 2016-02-29

**Authors:** Fabia U. Battistuzzi, Kristan A. Schneider, Matthew K. Spencer, David Fisher, Sophia Chaudhry, Ananias A. Escalante

**Affiliations:** Department of Biological Sciences, Oakland University, Rochester, MI USA; Department of MNI, University of Applied Sciences Mittweida, Mittweida, Germany; Department of Geology and Physics, Lake Superior State University, Sault Ste. Marie, MI USA; David Eccles School of Business, University of Utah, Salt Lake City, UT USA; Center for Molecular Medicine and Genetics, Wayne State University, Detroit, MI USA; Institute for Genomics and Evolutionary Medicine, Temple University, Philadelphia, PA USA

**Keywords:** Low complexity regions, Apicomplexa, Repetitive regions, Homopolymers, Complexity threshold, *Plasmodium falciparum*, Composition bias

## Abstract

**Background:**

Low complexity regions (LCRs) are a ubiquitous feature in genomes and yet their evolutionary history and functional roles are unclear. Previous studies have shown contrasting evidence in favor of both neutral and selective mechanisms of evolution for different sets of LCRs suggesting that modes of identification of these regions may play a role in our ability to discern their evolutionary history. To further investigate this issue, we used a multiple threshold approach to identify species-specific profiles of proteome complexity and, by comparing properties of these sets, determine the influence that starting parameters have on evolutionary inferences.

**Results:**

We find that, although qualitatively similar, quantitatively each species has a unique LCR profile which represents the frequency of these regions within each genome. Inferences based on these profiles are more accurate in comparative analyses of genome complexity as they allow to determine the relative complexity of multiple genomes as well as the type of repetitiveness that is most common in each. Based on the multiple threshold LCR sets obtained, we identified predominant evolutionary mechanisms at different complexity levels, which show neutral mechanisms acting on highly repetitive LCRs (e.g., homopolymers) and selective forces becoming more important as heterogeneity of the LCRs increases.

**Conclusions:**

Our results show how inferences based on LCRs are influenced by the parameters used to identify these regions. Sets of LCRs are heterogeneous aggregates of regions that include homo- and heteropolymers and, as such, evolve according to different mechanisms. LCR profiles provide a new way to investigate genome complexity across species and to determine the driving mechanism of their evolution.

**Electronic supplementary material:**

The online version of this article (doi:10.1186/s12862-016-0625-0) contains supplementary material, which is available to authorized users.

## Background

Repetitive regions have been ubiquitously found in all genomes analyzed so far, making them a common comparative feature for genome complexity. These regions belong to a larger category of sequences, known as low complexity, which are characterized by low diversity in residues, either nucleotides or amino acids. Within the broad definition of low complexity regions (LCRs) are included sequences that differ in terms of their level of repetitiveness (periodic or aperiodic motifs) and of their composition (homo- or heterogeneous) but that share an overall low diversity of residues compared to flanking (also known as background or high complexity) regions [1]. Because of the heterogeneity of LCRs, regions within this category are often sub-categorized according to their unique structure and composition producing a large terminology associated with LCRs that includes micro/mini-satellites, tandem repeats, interspersed repeats, simple sequence repeats, single amino acid repeats, homopolymers, and heteropolymers. While some of these categories apply to both nucleotide and amino acid LCRs, others are specific to one or the other residue type and are, therefore, applicable to specific questions. For example, microsatellites and LCRs in non-coding regions are often used as genetic markers to investigate polymorphisms on a short evolutionary timescale while LCRs within protein-coding regions bear the imprint of evolutionary processes over long timescales and can be informative on functional roles of these regions within a protein.

Among eukaryotes, *Plasmodium falciparum* and *Dictyostelium discoideum* are known to have some of the highest levels of genes harboring low complexity regions (LCRs) (at least 50 %) compared to other eukaryotic model organisms (~10–20 %) [2], which raises the question of what the underlying causes are that regulate the presence of these regions within genomes.

The LCRs of *P. falciparum* in particular have been widely studied in an effort to decipher a possible connection between the variability of these fast evolving sequences and some phenotypes, such as evasion of the host immune response that could confer an adaptive value. These efforts have focused primarily on the characterization of the composition, frequency, and evolutionary mechanisms of these regions in protein-coding genes [3–6] but have achieved contrasting results. While the composition of these regions is not debated (high prevalence of asparagines, N), their frequency and evolutionary mechanisms are less clear. For example, previous studies have proposed a wide range of frequencies of genes harboring LCRs (50–90 %) that depend on the thresholds used to identify these regions [2, 7, 8], which lead to the formation of overlapping but non-identical LCR sets. These different LCR groups have been found to be evolving both neutrally and by selection, but the reasons for this contrasting results are unclear [5–11]. A recent hypothesis focused on the compositional heterogeneity within a single set of LCRs showing that GC-rich regions, heterogeneous LCRs with aperiodic motifs, and polyN regions evolve according to different mechanisms [9]. This initial result suggests that the composition of LCR sets and, therefore, their mode of identification may play a larger role in our understanding of their evolution than previously thought.

Current methods to identify LCRs are based on parameters that include the length of the region (window size) and a threshold that determines their status as “low complexity” (complexity threshold). The values of these parameters are chosen depending on the regions that are of interest and are, therefore, dependent on the context of each study; for example, a study of single amino acid repeats will use a complexity threshold that will identify LCRs composed of just one amino acid repeated multiple times. However, despite the subjectivity of these parameters, inferences from specific LCR sets have been applied on a large scale to a variety of genomes, thus expanding their applicability beyond the study that originally identified them. The implicit assumption of this approach is that LCRs are regions that are intrinsically distinct from their genetic surroundings and can, therefore, be identified by an absolute measure (e.g., complexity threshold). Unfortunately, there is currently no evidence to support this view but rather it is equally possible that LCRs exist in a relative state and their properties should be defined based on a comparative measure of their complexity relative to that of the rest of the gene or genome. In this case, metrics that take into account the compositional bias of the genetic background in which the LCR is embedded should be developed.

To gain a comprehensive picture of LCRs in genomes, we performed a set of analyses that estimate the overall complexity of genomes in fully-sequenced Apicomplexa species using a multiple threshold approach. This approach produces profiles of frequencies of LCRs that are summarized by two objective parameters (derived from the profile curve) and that can be used to evaluate the presence of different low complexity categories and test hypotheses on their evolution. We chose the Apicomplexa as an example because of the large amount of information already available for *P. falciparum*, the number of related genomes available, and the wide range of compositional bias in the genomes of these species, which allowed us to explore the correlation between LCR sets and their evolutionary history. In particular, we focus on two aspects: first, a comparison of the nature of LCRs identified by a single and multiple thresholds and, second, the consequences of using single-threshold datasets to determine the evolutionary history of these regions. We chose to focus on LCRs in protein-coding regions rather than nucleotides to reduce the saturation bias accumulated by nucleotide sequences over long evolutionary time frames and that we would expect to be particularly strong in LCRs that are known to evolve with fast evolutionary rates [9, 12]. We do, however, use overall nucleotide compositions of genomes to investigate relations between LCRs and genome compositional biases.

Using proteome-wide approaches we find that the shape of the distributions of the complexity profiles (as determined by the frequency of LCRs at multiple thresholds) of all apicomplexans is comparable and can be described by a linear regression on the logits of the frequency of the LCRs. However, we also find that the abundance of LCRs is species-specific and independent from the composition bias of genomes and phylogenetic history. We also find that evolutionary mechanisms of these regions are correlated to the level of complexity of the regions themselves with homopolymers evolving predominantly by neutral mechanisms and selection acting more strongly as heterogeneity increases. Our results show the importance of using a multi-threshold approach in the identification of LCRs and the risks of generalizing trends across species, even if closely related.

## Results

The most commonly used method to identify low complexity regions is the software SEG that implements Shannon’s entropy to calculate the amount of information within a segment (or window, W) of a sequence. Whether implemented within SEG or used independently, Shannon’s entropy is the most widely used measure of complexity of a string of characters, such as a sequence [13, 14]. However, measuring the complexity of a sequence is not sufficient to identify LCRs as these are defined in a comparative framework as being “less” complex than other regions. Thus, any LCR-detection method inevitably depends on a threshold to distinguish low vs. high complexity. In current methods this threshold (here referred to as the complexity threshold, K) is user-defined and, therefore, subjective. For example, the choice of parameters in SEG depend on how conservative the user wants to be in identifying sets of low complexity regions: large window sizes and low complexity values will identify fewer regions than parameters for shorter, more diverse sequences. This absence of “optimal” parameters often results in the use of default or standardized values that bias the characteristics of the LCRs identified and the downstream inferences on their evolutionary and functional roles.

**Fig. 1:**
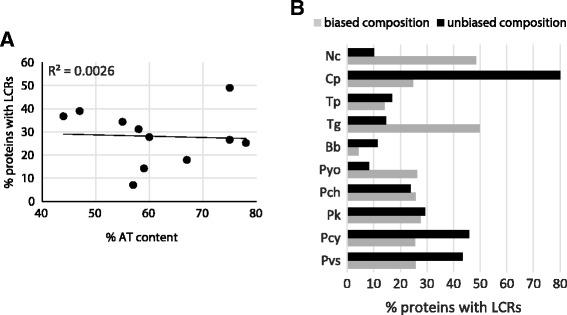
Relation of proteome AT content with frequency of LCRs calculated at *K* = 1.9. **a** Overall AT content is calculated based on the proteome of each species. **b** Trends in AT-rich/poor (biased) and AT-balanced (unbiased) proteins. Boundaries for nucleotide enrichment were < 45 % and > 55 %. Trends were comparable when < 40 % and > 70 % boundaries were used. Species abbreviations are as in Table 1

**Fig. 2:**
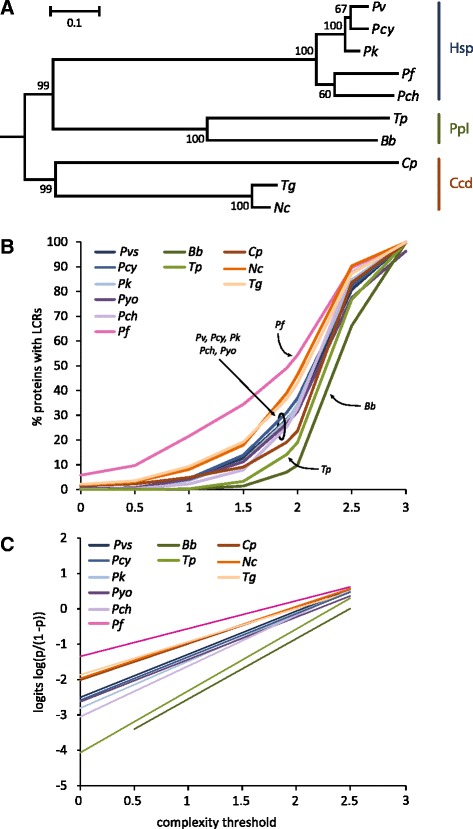
Species-specific frequency of LCRs for multiple complexity thresholds in Apicomplexa. **a** Maximum likelihood phylogenetic tree of Apicomplexa species used for the phylogenetic contrast analysis. *Pyo* was excluded from this analysis because it lacked chromosome assignments for its proteins, which was necessary for the phylogenetic contrast analysis. The phylogeny was obtained using 30 orthologous genes randomly selected from Kuo et al. (2008). Bootstrap values are shown at each node. Species belonging to the same taxonomic group are shown (Hsp: Haemosporidia, Ppl: Piroplasmida, Ccd: Coccidia). **b** LCRs profiles with complexity thresholds = 0–3 (color coding refers to individual species and taxonomic groups: blue/purple: HSP; green: Ppl; Orange/brown: Ccd). **c** Linear regression on the logits of LCR frequencies. Values at *K* = 3 were excluded because virtually indistinguishable from the background composition

While initial analyses of genome complexity were carried out with the SEG default parameters (*W* = 12, *K* = 2.2), more recent studies suggested the use of *W* = 15 and *K* = 1.9 for eukaryotes to identify longer and more repetitive low complexity regions [15, 16]. We started our analysis based on these parameters and identified LCRs in representative Apicomplexa genomes, which show a continuum of frequencies of LCRs ranging from low (<20 %) in *Babesia bovis (Bb), Theileria parva (Tp),* and *Cryptosporidium parvum (Cp)*, to medium (>20 % but < 40 %) in *Plasmodium vivax (Pv), Plasmodium cynomolgi (Pcy), Plasmodium knowlesi (Pk), Plasmodium yoelii (Pyo), Plasmodium chabaudi (Pch), Neospora caninum (Nc),* and *Toxoplasma gondii (Tg)*, to high (>40 %) in *Plasmodium falciparum (Pf)* (Table 1). Interestingly, this pattern does not follow overall genome composition bias measured as the dependency of AT content of the protein-coding genes and the frequency of the LCRs (Kendall rank correlation: −0.26; 95 % bootstrap confidence interval: −0.72–0.39) (Fig. 1a). Even when we analyzed the frequency of LCRs in compositionally biased and unbiased proteins individually, we found that for most species LCRs are more frequent, or equally present, in unbiased proteins, suggesting that the overall composition bias of a protein is not a determining factor in the formation of LCRs (Fig. 1b). Exceptions to this trend are *Nc, Pyo,* and *Tg* in which LCRs are preferentially found in biased proteins. Additionally, the frequency of LCRs in each genome does not show any influence from phylogenetic relationships as assessed by a phylogenetic contrast analysis (*p*-value = 0.16; see Methods) carried out by evaluating the correlation between LCR frequency and phylogenetic relatedness for each chromosome in each species (*Pyo* was excluded from this analysis because its proteins could not be separated into different chromosomes at the time of the analysis) (Fig. 2a).

**Table 1:** Statistics of 11 apicomplexa genomes

Tax. group	Genome	# of chr	Protein coding genes	AT (%)	S_proteome_	% LCR frequency (*K* = 1.9)	*S*_%_ (*K* = 1.9)	% LCR frequency (*K* = 1)	*S*_%_ (*K* = 1)
Hsp	*Pv*	14	5435	55	16.62	34.3	19.9	5.08	26.96
	*Pcy*	14	4988	58	16.45	31.1	56.64	4.5	27.5
	*Pk*	14	5122	60	16.36	27.72	55.96	3.67	28.56
	*Pyo*	14	7724	75	13.71	26.5	40.6	4.16	16.06
	*Pch*	14	5042	78	14.05	25.2	53.15	2.27	25.86
	*Pf*	14	5410	75	13.35	49	31.88	2.16	6.2
Ppl	*Bb*	4	3706	57	17.23	7	59.32	0.16	29.92
	*Tp*	4	4082	59	16.25	14.2	71.59	0.34	39.51
Ccd	*Cp*	8	3805	67	15.68	19.5	62.33	5.39	33.61
	*Nc*	14	7080	47	14.51	39	37.74	8.15	9.83
	*Tg*	14	8102	44	14.75	36.7	36.49	9.34	8.83

LCRs are identified using a window size of 15 and a complexity threshold (K) of 1.9 and 1 as examples. LCRs frequency: percentage of proteins with at least one LCR. S_%_: Simpson’s Reciprocal Index relative to the diversity of the proteome. The AT content is calculated from the proteome of each species

*Abbreviations*: *Tax* Taxonomic, *Hsp* Haemosporidia, *Ppl* Piroplasmida, *Ccd* Coccidia, *Pv Plasmodium vivax, Pcy P. cynomolgy, Pk P. knowlesi, Pyo P. yoelii, Pch P. chabaudi, Pf P. falciparum, Bb Babesia bovis, Tp Theileria parva, Cp Cryptosporidium parvum, Nc Neospora caninum, Tg Toxoplasma gondii*, *chr* chromosomes, *LCRs* low complexity regions

This single threshold view informs us on relative complexities across species and can lead to generalized conclusions such as *Cp* having a proteome that is more complex (i.e., fewer LCRs) than that of the plasmodia (Table 1). However, a different threshold would produce different results (e.g., *K* = 1 in Table 1) because the single threshold approach does not take into account the possibility that genomes might have different types of low complexity regions. To explore this possibility, we expanded this analysis to include multiple levels of complexity (keeping the window size at 15 residues) in increments of 0.5 from 0 to 3 (we refer to this method as multi-threshold). This approach allowed us to reconstruct profiles of the Apicomplexa genomes that effectively describe the complexity of a genome using two parameters: the baseline repetitiveness of each genome identified by the amount of homopolymeric regions (y-intercept) and the genome-specific response to increasing complexity (slope) (Fig. 2). The trends of this species-specific profiles can then be used to objectively describe the complexity level within a genome and illuminate the effects that parameters used to identify low complexity regions will have on the produced sets. For example, it is clear that each genome starts from a unique frequency of homopolymeric regions but all genomes show a fast increase in LCRs for thresholds K between 1.5 and 2.5. Higher thresholds produce slower increments of proteins with LCRs eventually reaching the maximum of at least one LCR in every protein (frequency of 100 %) at *K* = 3 in most species. The observed plateauing in the rate of LCR increase suggests that the distinction between LCRs and background genome composition is breaking down therefore questioning a biological significance of LCRs at these levels of complexity and providing an upper bound for LCR detection. Despite the similarity in trends, the profiles show unique overall amounts of repetitiveness in each genome, as shown by the significantly different slopes of the log-linear regressions (ANCOVA *p*-value: <<0.001) (Fig. 2b). In other words, qualitatively all genomes have similar profiles for LCR frequency but they differ quantitatively. These analyses were carried out with a fixed incremental value for the window extension parameter (K_2_; see Methods) used by SEG. We investigated the effect that K_2_ might have on the identification of LCRs and found that more permissive K_2_ values promote the merging of multiple LCRs into a single one. However, we find that these effects reduce the total number of LCRs by an average of only 11 % (8–18 %) for a large range of values (*K* = 1.9, K_2_ ranging from 1.9 to 3). Moreover, for each K value, different species have patterns of LCR changes that are similar (e.g., at *K* = 2 *Pf* and *Pvs* have a 10 % reduction of LCRs), which means that the relative comparisons of LCR profiles among species is unaffected by K_2_.

At a higher taxonomic level, the three major Apicompexa groups, Haemosporidiae (*Pv, Pcy, Pk, Pyo, Pch, Pf*), Piroplasmida (*Bb, Tp*), and Coccidia (*Cp, Nc, Tg*), show different complexity profiles with an increasing frequency from Pirosplasmida to the Coccidia. Both *Tp* and *Bb* are severely depleted in LCRs with very low frequencies (0.03 % for *Tp* and 0 % for *Bb*) of homopolymeric regions. Within Plasmodia (Haemosporidiae), all genomes except *Pf* show intermediate LCR frequencies followed by the Coccidia (*Nc,* and *Tg* in particular) and by *Pf* (Fig. 2). The large range of LCR profiles observed among species does not seem to be related to taxonomic or phylogenetic clustering nor does it reflect compositional biases in the proteome of these species. Indeed lineages with similar AT contents (whether high AT in *Pf, Pyo* and *Pch*: 75–78 %; or intermediate AT in *Pv, Pcy, Pk, Bb*, and *Tp*: 55–60 %) have widely different profiles.

In addition to the frequency of LCRs in proteomes we also estimated their composition relative to their background genomes, which provides a way of evaluating their uniqueness within proteins. To do this we used a modified version of the Simpson’s Reciprocal Index to measure the overall difference in amino acid usage (i.e., diversity) between the proteome and LCRs identified by different complexity thresholds (S_%_; see Methods). When applied to LCRs of different species detected with *K* = 1.9, we find that the diversity of these regions is highly variable among species ranging from *S* = 32 % to *S* = 72 % (Table 1), which means that regions identified with the same complexity threshold are more (32 %) or less (72 %) unique compared to their proteomes. Within the multi-threshold framework, all species increase in diversity with increasing complexity, as expected, reaching values between 60 and 86 % for *K* = 2.5. Also by this measure, *Pf* has a unique trend being the species with the lowest LCR diversity value across all complexity thresholds, which reflects its strong preference for asparagine use in LCRs.

Finally, we used the LCR profiles obtained by the multi-threshold approach, to test the effect that LCRs’ composition has on our ability to discriminate between neutral and selective evolutionary mechanisms. In the latter scenario, no attempt is made to distinguish between positive and purifying selection but rather selective mechanisms are identified based on non-random outcomes of LCR evolution. To identify these processes we followed previous methods and compared the frequency of LCRs to the compositional bias of the proteome in each species and also compared the empirical location of the LCRs within proteins against a modeled random distribution [17]. In these comparisons, neutral evolution would be supported by a strong correlation between LCR frequency and AT content of the proteome and a random distribution of empirical LCR locations. We found evidence against both these hypotheses with no dependency between LCR frequency and AT composition (Kendall’s rank correlation not significantly different from 0; see Additional file 1) and non-random distributions for heterogeneous LCRs (Table 2). In particular, we find that for most Apicomplexa species a threshold of ~1.9 and higher corresponds to non-random distributions of these regions within proteins, therefore suggesting an active selective pressure.

**Table 2:** Distribution of single low complexity regions in proteins

Tax. group	Sp.	*K* = 0	*S*_%_ (*K* = 0)	*K* = 0.5	*S*_%_ (*K* = 0.5)	*K* = 1	*S*_%_ (*K* = 1)	*K* = 1.5	*S*_%_ (*K* = 1.5)	*K* = 1.9	*S*_%_ (*K* = 1.9)	*K* = 2.5	*S*_%_ (*K* = 2.5)
Hsp	*Pv*	R (23)	0^a^	R (47)	10.38	R (240)	26.96	R (601)	43.53	R (995)	56.79	**NR (976)**	**76.68**
	*Pcy*	R (20)	0^a^	R (33)	11.30	R (196)	27.49	R (497)	43.69	R (893)	56.64	**NR (1019)**	**76.07**
	*Pk*	**NR (9)**	**0** ^a^	R (31)	13.33	R (154)	28.56	R (447)	43.78	**NR (890)**	**55.96**	**NR (914)**	**74.23**
	*Pyo*	R (21)	2.30	R (57)	7.40	R (286)	16.06	R (647)	28.27	**NR (1208)**	**40.60**	**NR (1480)**	**63.35**
	*Pch*	R (7)	0^a^	R (18)	10.70	R (102)	25.86	R (308)	41.02	**NR (746)**	**53.15**	**NR (764)**	**71.35**
	*Pf*	R (77)	1.50	**NR (406)**	**0.72**	**NR (693)**	**6.20**	**NR (876)**	**17.96**	R (978)	31.88	**NR (654)**	**60.29**
Ppl	*Bb*	NC	NC	NC	NC	R (6)	29.92	R (51)	46.26	**NR (210)**	**59.32**	**NR (1068)**	**78.93**
	*Tp*	NC	NC	NC	NC	**NR (13)**	**39.51**	R (124)	58.74	R (434)	71.58	**NR (964)**	**86.64**
Ccd	*Cp*	R (56)	0^a^	R (78)	15.62	R (152)	33.61	**NR (240)**	**50.15**	**NR (431)**	**62.33**	**NR (811)**	**78.87**
	*Nc*	R (111)	0^a^	**NR (191)**	**1.58**	**NR (453)**	**9.84**	**NR (825)**	**23.27**	**NR (1284)**	**37.74**	**NR (894)**	**65.66**
	*Tg*	**NR (141)**	**0** ^a^	**NR (243)**	**1.18**	**NR (574)**	**8.83**	**NR (968)**	**21.99**	**NR (1416)**	**36.48**	**NR (1313)**	**64.85**

*R* random, *NR* non random (bold), *K* complexity threshold, *S*_*%*_ diversity at K relative to the proteome diversity. In parenthesis are shown the total number of proteins considered in each case. Genome abbreviations are as in Table 1. *NC* not computable

^a^These values have been forced to zero where S_%_ is negative due to lower accuracy of the fitted equations at low complexity

## Discussion

Interest in the evolution and function of eukaryotic LCRs is growing, especially since their associations with diseases and other phenotypic modifications (e.g., antigenic variability) were discovered [1, 6, 18–26]. Comparative analyses of low complexity regions within proteins allow for the identification of changes that may affect protein functionality, revealing possible selection-driven roles of these regions [27–29]. Non-random patterns of changes within LCRs are providing increasingly strong evidence for selective pressures acting on LCRs, in opposition to previously hypothesized neutral models of their evolution [7–11, 17, 27, 28, 30–41]. However, despite the large amount of information that is being collected on these regions in multiple species, little is known of the effects that the parameters used to identify these regions are playing on the inferred evolutionary and functional mechanisms. To investigate this aspect, we obtained LCR profiles of apicomplexan genomes spanning a continuum of complexity levels in order to compare inferences drawn from a single-parameter and a multi-parameter view of proteome complexity. All our profiles are based on a window size of 15 residues that is a user-specified parameter widely used in previous studies. While we primarily focus on this window size as it allows us to directly compare ours and previous results, we also explored how low complexity profiles change with smaller and larger windows (6 and 24). As expected, the biggest impact of window size is on the total number of LCRs detected, which is larger for smaller windows. Therefore, plateauing of the sigmoidal profile for *W* = 6 is reached at lower complexity thresholds compared to *W* = 15 and it is never reached at *W* = 24. In addition to these values, we carried out an initial analysis with multiple window sizes around the value of 15 commonly used (*W* = 12 through 18) and have found no significant difference in species-specific profile trends between *W* = 15 and the other windows (for each species *p*-value > 0.05 in a multiple linear regression analysis). Therefore, the results obtained in this study with *W* = 15 are likely to be applicable to a variety of scenarios. However, because larger changes in window size (*W* = 6, 24) produce significantly different LCR profiles, more analyses are required to determine the role of W.

Our results show that all apicomplexan genomes are repetitive in nature but that the type of repetitiveness is species-specific. For example, some genomes (e.g., *Bb*) lack homopolymeric regions but still have other types of less repetitive heteropolymers (frequency of 66 % for LCRs at *K* = 2.5). This suggests that conclusions on the overall complexity of a genome drawn and generalized from a single threshold approach might be misleading. The scenario of LCRs defined by *K* = 1.9 confirms previous findings of high frequency of these regions in *Plasmodium falciparum* (49 %) but also show that these results are not shared by other plasmodia and Apicomplexa in general, which have lower frequencies (7–39 %). Such observations can lead to generalized conclusions that may or may not be accurate. For example, based on this result is it accurate to conclude that other apicomplexan genomes are, overall, less repetitive than *Pf*? To answer this question we used a multi-threshold approach to estimate LCRs. When we compare patterns of LCR frequencies obtained with this approach it is clear that depending on the complexity level under consideration the answer changes. For example, in the case of *Pf*, the multi-threshold approach confirms its uniqueness as its genome is consistently higher in frequency of LCRs irrespective of the complexity level used. However, other genomes show different patterns depending on the complexity threshold, such as *Pch* that is less complex than most plasmodia for *K* < 1.9 but becomes more complex at higher K values (Fig. 2b).

While frequencies estimate the prevalence of LCRs in genomes, composition is used to evaluate their potential functional importance. Factors that are generally considered are the type of repetitiveness within LCRs and their residue composition as they play a role in 3D protein structures and hydrophilicity/hydrophobicity of specific regions within a protein [2, 32, 42–46]. Moreover, the relation between the amino acid composition of LCRs and the compositional bias of their background genome can provide clues to the evolution of these regions, as genomes that have similar compositional biases would be expected to have similar LCR residue preferences under a neutral model of evolution. To explore this possibility, we developed a new application of the ecological Simpson’s reciprocal index, the diversity index, that allowed us to correlate the composition of LCRs and their native genomes. Using this metric we find that LCRs identified based on the same complexity threshold are composed of regions that are widely diverse (i.e., different number of amino acids represented within the region) relative to their background proteome, such as in the case of *K* = 1.9 that identifies LCRs with diversity indices ranging from 32 % (*Pf*) to 72 % (*Tp*) (a value of 100 % would mean that the total number of amino acids used in the LCRs is equal to that used in the overall proteome). Conversely, LCRs that have similar diversity indices have very different complexity thresholds suggesting that LCR sets identified taking into account the compositional bias of the native genome will differ from those obtained with absolute measures, whether based on single or multiple thresholds.

**Fig. 3:**
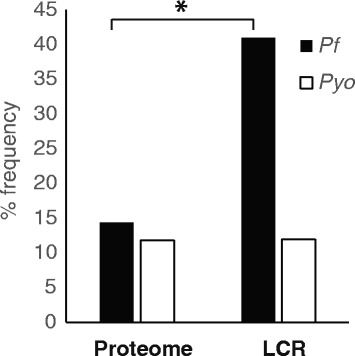
Usage of the most common amino acid (asparagine) in two species with similar AT content (*Pf* and *Pyo*). Significant (*p*-value < 0.001) preference for Asparagine usage in LCRs is shown by the asterisk

The wide range in diversity observed within LCRs estimated with both absolute and relative measures suggests a preferential usage of amino acids in these regions that differs from that of the proteome. This hypothesis can be explored by comparing the diversity levels of LCRs in genomes with similar compositional biases that, under a neutral model of evolution, would be expected to preferentially select the usage of the same amino acids based on their codon composition. We find that this is not true as *Pf* and *Pyo*, both with a 75 % AT content, vary in their diversity (*S*_%_ = 32 and 41, respectively). Moreover, in *Pf* the amino acid asparagine is preferentially used in LCRs (41 %) compared to the background proteome (14 %) (Grubbs’ test *p* < 0.001) while in *Pyo* it is not (Fig. 3). This difference cannot be explained by variance within regions of the proteome as a bootstrap analysis shows that the asparagine frequency in LCRs is not included in the 95 % interval of the proteome variance. Asparagine is one of the amino acids encoded by AT-rich codons along with Lysine, Tyrosine, Leucine, and Isoleucine so, under a neutral model, each of these amino acids would be expected in the LCRs at a similar ratio as that in the proteome. We do not observe this in the case of *Pf* and *Pyo* as both have a strong preference in their LCRs for only one of the AT-rich amino acids (Asparagine and Isoleucine, respectively). Since this behavior cannot be explained by compositional biases alone, it suggests the action of selection in determining the composition of LCRs. It will be interesting in the future to investigate if the relative diversity of LCRs can be used as a reliable predictor for functional or structural importance.

Finally, we used the LCR profiles of complexity and diversity to determine a possible role of selection in the evolution of LCRs by comparing their empirical location within genes to an expected distribution under a random (neutral) model of evolution. Our main aim for this analysis was to compare the results from the single and multiple threshold approach to identify possible biases intrinsic to the set of LCRs. Using the single threshold approach with *K* = 1.9 we find that most of the species analyzed show a non-random distribution of LCRs suggesting that selection is acting on these regions (*Pk, Pyo, Pch, Bb, Cp, Nc, Tg*) (Table 2). Interestingly, *Pf* is not included among these species although the previous and following complexity thresholds show LCRs as being under selection. To investigate this result we performed a composition-based analysis of the LCRs in *Pf* following a recent study (based on a single threshold) that showed how LCRs in *Pf* might be evolving under different mechanisms depending on their composition (e.g., High GC LCRs were shown to be under selection) [9]. Under this scenario, the LCRs under selection would be expected to show a preferential use of GC-encoded amino acids. We compared the amino acid usage of LCRs in the three complexity categories that differ in their evolutionary mechanism (*K* = 1.5, 1.9, and 2.5) to investigate this possible preferential use of GC-rich amino acids for *K* = 1.5 and 2.5 compared to the amino acids at *K* = 1.9. We found no evidence of higher GC-encoded amino acids in the LCRs under selection leaving the result of the seemingly neutral evolution of LCRs from *K* = 1.9 unexplained. It should be noted that, if proteins with any number of LCRs in them were considered, the location of LCRs in *Pf* at *K* = 1.9 was not random. This result could suggest that the observed randomness of single LCRs could be a false negative result caused by the smaller number of proteins considered (978 with single LCRs vs. 9184 with single and multiple LCRs).

Irrespective of the evolutionary behavior of LCRs in *Pf*, it is clear that not only the evolutionary mechanism based on a single-threshold selection for LCRs cannot be generalized to all species, it should also not be applied to all categories of LCRs within a species. Indeed, when we use a multi-threshold approach we find that as regions become less complex their distribution within proteins is more likely to follow a random pattern with most species having random LCRs if identified by complexities lower than 1.9 (Table 2). A similar result was found using the diversity index, with more diverse LCRs (higher S_%_) more likely to be under selection than less diverse regions. Therefore, these results suggest that the current approaches to study the evolutionary mechanisms of LCRs estimated with a single-threshold approach (e.g., *K* = 1.9) will promote a selection-driven view of the evolution of these regions that is not applicable to all complexity levels.

Our results show a connection between level of complexity and evolutionary mechanisms for LCRs that has been undetected. the multi-threshold scenario suggests that in most Apicomplexa, regions within a protein that start as homopolymers are most likely to evolve neutrally and, therefore, accumulate changes (i.e., progressively increase their complexity) that might eventually lead to the gaining of a functional role. Once this happens, selective pressures become the driving force in their evolution thus shifting the signal that we can detect with genome-wide analyses from neutral to selection-driven evolution. Interestingly, we find that in a few species (*Pf*, *Nc*, *Tg*) these regions seem to be evolving under selection irrespective of their complexity status, which raises the possibility that LCRs in these species might be functionally different from other LCRs. Overall, we find that the contrasting results on the evolution of LCRs obtained by previous studies could be caused not only by heterogeneous sets of regions but also by signals from multiple evolutionary mechanisms that change through time and across species [4–7, 9–11].

## Conclusions

Multi-threshold profiles of LCRs provide a new outlook on genome complexity that allows objective genome-wide comparisons across species. The multi-threshold approach shows LCRs as an emergent property of Apicomplexan genomes that is unrelated to the phylogenetic history and compositional bias of the species. It also shows that LCRs evolve under different mechanisms (selection or neutral) based on their complexity and diversity. The correlation between LCR complexity and evolution can explain the difficulties in identifying a unifying evolutionary principle for these regions and support the use of comprehensive profiles to investigate the evolution of genome complexity.

## Methods

### Identification of LCRs

We analyzed 11 representative Apicomplexa genomes obtained from the publicly available databases PlasmoDB (v. 8.2), CryptoDB (v. 4.6) and EupathDB (v. 2.13): *Plasmodium vivax Salvador-I (Pv), P. cynomolgi strain B (Pcy), P. knowlesi strain H (Pk), P. chabaudi chabaudi (Pch), P. yoelii yoelii 17XNL (Pyo), P. falciparum 3D7 (Pf), Babesia bovis T2Bo (Bb), Theileria parva strain Muguga (Tp), Cryptosporidium parvum Iowa II (Cp), Neospora caninum (Nc)*, and *Toxoplasma gondii GT1 (Tg)*. For each genome we identified low complexity regions in their proteins using the algorithm implemented in the program SEG [13, 47]. This program identifies and extracts low complexity regions within a protein according to two user-specified thresholds (K_1_ and K_2_). Complexity of regions of length equal to the specified window size (e.g., 12) is calculated and compared to the first user-defined threshold (K_1_): if lower, they are reported as low complexity regions and the window is extended on both sides as long as the complexity of the extended regions remains lower than K_2_. For the single threshold approach, we used fixed values of *W* = 15 and *K*_1_ = 1.9 as has been done previously [15, 17]. For the multi-threshold approach, we used a variety of parameter combinations: for the window size parameter we halved and doubled the default value in SEG (6 and 24), used a window of 15 sites, and also repeated the analyses with *W* = 12–18. Then, for the three main window sizes (6, 24, and 15), we investigated the effect of the complexity threshold (K_1_) by altering this parameter from 0 to 3 at 0.5 intervals (Additional file 2). For *W* = 15, we also altered K_1_ at 0.1 intervals and found comparable trends. We tested the effect of K_2_ by increasing it from 0.1 to 3.3 in 0.1 steps. Because the changes in LCR distribution within proteomes caused by different K_2_ values did not affect the trend of the frequency of LCRs we calculated for each K_1_ a value of *K*_2_ = K_1_ + 0.3 as suggested by default and used previously [7, 9, 27]. In the results and discussion section we refer to K_1_ as K. Initial analyses showed evidence that LCRs at *K* = 3 are not significantly different from the proteome composition as evidenced by the presence of LCRs in 90–100 % of the proteins; therefore all analyses are conducted with K values up to 2.5.

### Frequency of LCRs

A plot of the percentage of proteins with LCRs vs. the complexity threshold (K_1_) showed a sigmoidal dependence of these two quantities. Therefore, we performed a linear regression on the logits of the LCR frequencies, i.e., if *p* is the frequency of LCRs, *logit p = log (p/(1-p))*. The linear regression was performed with SAS v. 9.2 for each genome separately [48]. For each linear regression, an F-test was performed on a 5 %-significance level to assess the model’s fit (tests for non-zero regression slope). Moreover, the coefficients of determination (R-square and adjusted R-square) were calculated to explore the proportion of explained variation. Deviations from zero of estimates of the regression line’s slope and intercepts were tested using a *t*-test at a 5 %-significance level. If *p(K*_*1*_*)* denotes the percentage of LCRs for a given K_1_ (the dependence on K_2_ is ignored), then *logit p(K*_*1*_) ≈ a + b*K_*1*_, where *a* and *b* are the estimates of the intercept and slope of the linear regression. The intercept, *a*, is the logit-percentage of homopolymeric LCRs (regions of low complexity composed by only one amino acid). The slope, *b*, specifies the changes in the frequency of detectable LCRs in response to increasing K_1_. The slope allows us to describe how identifiable LCRs are against the complexity of the background for a given genome within a range of K_1_ values in this case but it could be applied to any other measure. Such observed low complexity functions are compared among genomes by an analysis of covariance (ANCOVA), with K_1_ as covariate. We also compared the logits obtained with window size equal to 15 with those obtained with window sizes between 12 and 18, 6, and 24 using a multiple linear regression analysis in R [49].

### Diversity of LCRs

As an additional measure of complexity, we used the Simpson’s Reciprocal Index (S) to estimate differences in amino acid compositions for LCRs with increasingly higher complexity thresholds and also for the full proteome. In ecology, this index is used to measure the species diversity in a biological community in terms of the probability of randomly sampling two individuals from the same species. In our investigation, we adapted it to use amino acids instead of species and proteomes instead of biological communities. Thus, we measure the probability of re-sampling the same amino acid by chance in LCRs defined at a given threshold $\left(D = {\displaystyle {\sum}_{i = 1}^{20}\frac{n_i\left({n}_i-1\right)}{N\left(N-1\right)}}\right)$ where *n*_*i*_ is the total number of amino acid *i* in all LCRs of a given genome and N is the combined length of all LCRs in the same genome and take the reciprocal of the Simpson’s Diversity index D (*S* = 1/D). We repeat this procedure also for the proteome and compare the spread of the diversity between the proteome and the subset defined by the complexity threshold to determine the variation in amino acid usage at these two levels (*S*_%_ = 100/((S_proteome_ – S_K0_)/S_K*i*_ – S_K0_))) where S_K0_ is the diversity for *K*_1_ = 0 and S_K*i*_ is the diversity at the complexity threshold *K*_1_ = *i*. Conversely, if we want to find the set of LCRs that collectively corresponds to a user-defined percentage (e.g., *S*_%_ = 25 %) of the amino acids diversity of the proteome, we calculate the complexity threshold that corresponds to S_%_ using a linear or quadratic best fit line (Additional file 3). Finally, to assess the significance of differences in the amino acid usage of proteomes and LCRs, we applied the Grubbs’ test to identify outliers within a normally distributed population. In our case, the population was composed of the differential frequencies of amino acid usage in LCRs compared to the proteome and was found to be normally distributed. We also created 2000 bootstrapped sets, and the corresponding 95 % confidence interval, from the proteome of the same size as the identified LCR set to measure the variance in amino acid usage within the proteome. We selected the Simpson’s index because it does not assume an equal probability of all categories or the presence of all categories in all groups, which – in the case of amino acids – would be violated (e.g., Cysteine is a much rarer amino acid than Asparagine). However, we also tried a second index in a subset of analyses, the Berger-Parker, and found similar trends.

### Tests of evolutionary mechanisms

To investigate the role of selection in the evolution of LCRs, we performed two sets of tests. First, we calculated the position of each LCR within each protein that was divided into three segments of equal length (N-terminus, middle, and C-terminus). Following Huntley and Clark [17], in the absence of selective constraints, we would expect LCRs to be randomly distributed within the protein based on the length of the LCR (l) and of the protein (L). The probability of the mid-point of an LCR falling into one of the three segments was calculated as follows: middle section (*L*/3)/(*L* − *l*); each of the termini (*L*/3 − *l*/2)/(*L* − *l*). This distribution was tested against the empirical distribution of LCR midpoints by a chi-square goodness-of-fit test. This simple null-hypothesis distribution implicitly assumes one LCR per protein, or that the protein length is large enough that the probability of two or more LCRs interfering with each other’s location in the same protein is negligible. However, many proteins harbor more than one LCR and, in some cases, their positions within a protein cannot be assumed to be independent from each other. Therefore, we performed the chi-square test only for proteins with single LCRs although this resulted, in a few cases, in datasets too small to resolve the test. To account for the small dataset sizes, we checked trends on unweighted and weighted chi-square results, with the last ones obtained by scaling the chi-square value to the number of genes in each category, and found similar patterns.

Second, we tested the neutral evolution hypothesis by comparing the frequency of LCRs to the compositional bias of each proteome and determine the dependency, or lack thereof, of these two parameters from each other as measured by the Kendall rank correlation coefficient [50]. We also calculated Spearman’s and Pearson’s coefficients and obtained identical results. We use simple bootstrap confidence intervals based on 2000 bootstrap replicates to check if the correlation coefficient between AT content and LCR frequency is significantly different from 0 [51]. To control for phylogenetic relationship, we carried out a phylogenetic contrast analysis performed using Mesquite and the PDAP module [52, 53]. For each genome, we analyzed each chromosome separately by calculating the chromosome AT content and LCR frequency. A total of 110 data points were obtained (14 chromosomes for *Pv*, *Pcy*, *Pk*, *Pch*, *Pf*, *Nc*, and *Tg*; 8 chromosomes for *Cp*; for *Bb* and *Tp* genes were assigned only to two of the four chromosomes). The genome of *Pyo* was not used because its proteins were lacking chromosome assignment information at the time of this study. The phylogeny and branch lengths used were obtained with a maximum likelihood (ML) tree-building method. The best protein substitution model (rtREV + F + G + I) was identified in MEGA v5.1 [54] and used to obtain the phylogeny. Thirty orthologous genes from Kuo et al. [55] were randomly selected, aligned and concatenated for our genomes. The orthologs for species missing from the original Kuo et al. dataset were added using OrthoMCL v.2.05 and MEGA [56]. The phylogeny obtained was comparable to that of Kuo et al. with the only difference being the clustering of *Cp* with *Tg* in our phylogeny ([55]; Fig. 2a). In order to carry out the phylogenetic contrast analysis, each lineage was divided into its chromosomes by assigning a virtually negligible branch length to each chromosome (0.00001). Inter-species branch lengths were obtained from the ML phylogeny.

Then we tested the correlation of LCRs with the AT content of the proteome overall and proteins categorized into two groups: compositionally biased and unbiased. Thresholds for the two groups were <45 % and >55 % for the biased proteins and between 45 and 55 % for the unbiased ones. We also repeated the analyses with threshold at 40 % and 70 % and found comparable results for the genomes that could be compared (species *Pv, Pk*, and *Bb* had too few proteins in the biased category). We then compared the number of LCRs in proteins belonging to these two categories. Although analyses were conducted on all genomes, a few of them (*Pk, Pyo, Tp, Cp*) were excluded because the number of proteins in the unbiased category was less than 10 % of the number of biased ones.

## Availability of data and materials

The datasets supporting the conclusions of this article are included within the article and its Additional files 1, 2, 3, and 4.

## Additional files

Additional file 1: Best fit trendlines between proteome AT content and LCR frequencies for increasing complexity thresholds. Each data point represents an apicomplexan species. Linear and quadratic best fits were tested with a bootstrap method (2000 replicates) and Kendall, Spearman, and Pearson’s correlations were estimated. All correlation show no significant dependency of the two parameters. (PDF 32 kb) Additional file 2: Profiles of LCRs with window sizes (W) of 6 and 24. Abbreviations are provided in the main text. (PDF 314 kb) Additional file 3: Species-specific best fit trendlines between complexity thresholds and the Simpson’s Reciprocal Index. Linear and quadratic best fit were tested with a bootstrap method (2000 replicates). Kendall, Spearman, and Pearsons’s coefficients show strong dependency of the two variables. Linear best fits are not shown for those cases where the quadratic best fit is not significantly different from 0. Abbreviations of species names are provided in the main text. (PDF 572 kb) Additional file 4: Accession numbers from PlasmoDB for each species. (XLSX 544 kb)
