# Supplementary figures and images for: Profiles of low complexity regions in Apicomplexa
Source: BMC Evol Biol. 2016 Feb 29;16:47. doi: 10.1186/s12862-016-0625-0 (PMC4770516; doi:10.1186/s12862-016-0625-0)

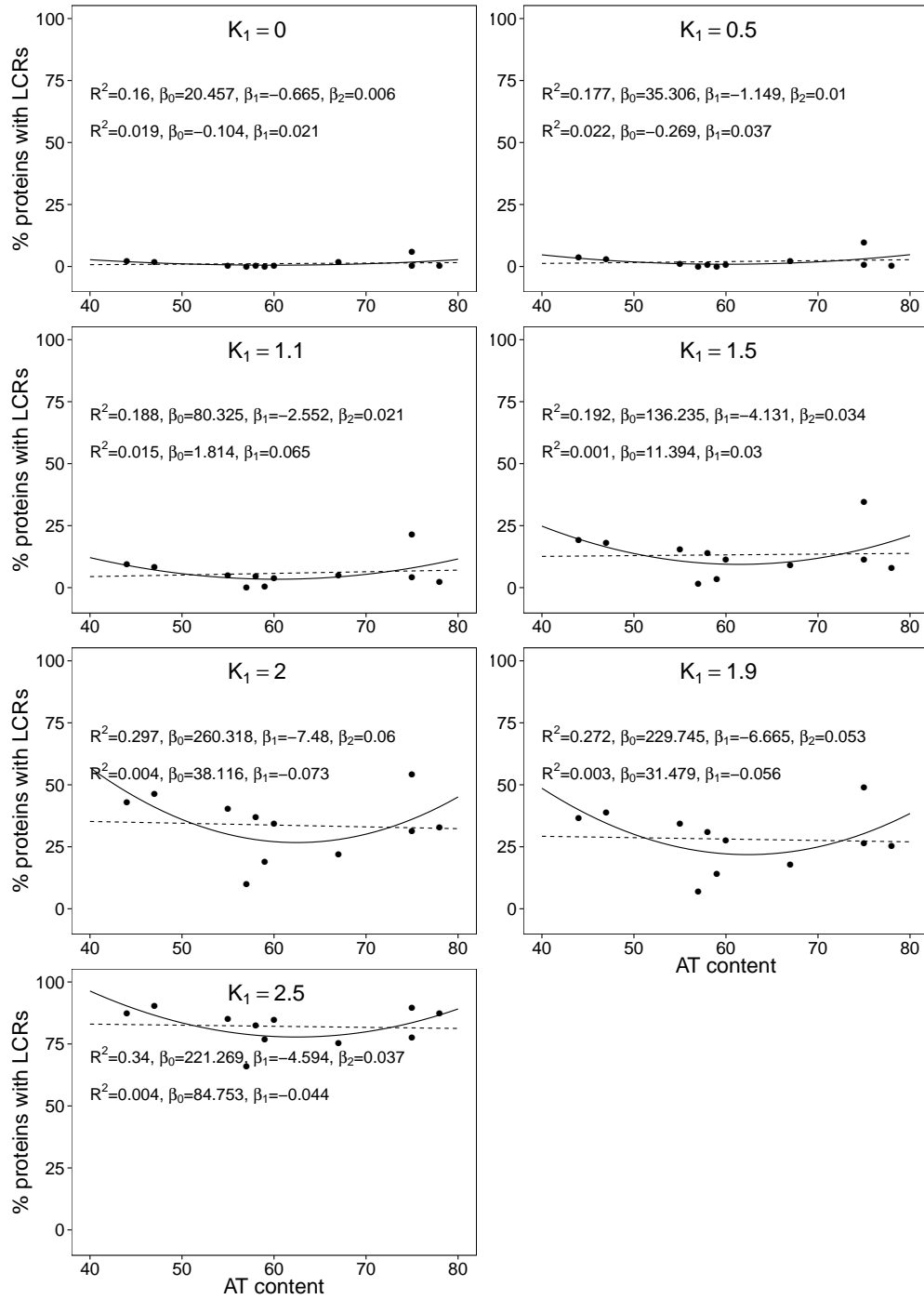

Supplement: Additional file 1: — Best fit trendlines between proteome AT content and LCR frequencies for increasing complexity thresholds. Each data point represents an apicomplexan species. Linear and quadratic best fits were tested with a bootstrap method (2000 replicates) and Kendall, Spearman, and Pearson’s correlations were estimated. All correlation show no significant dependency of the two parameters. (PDF 32 kb) [file 12862_2016_625_MOESM1_ESM.pdf]

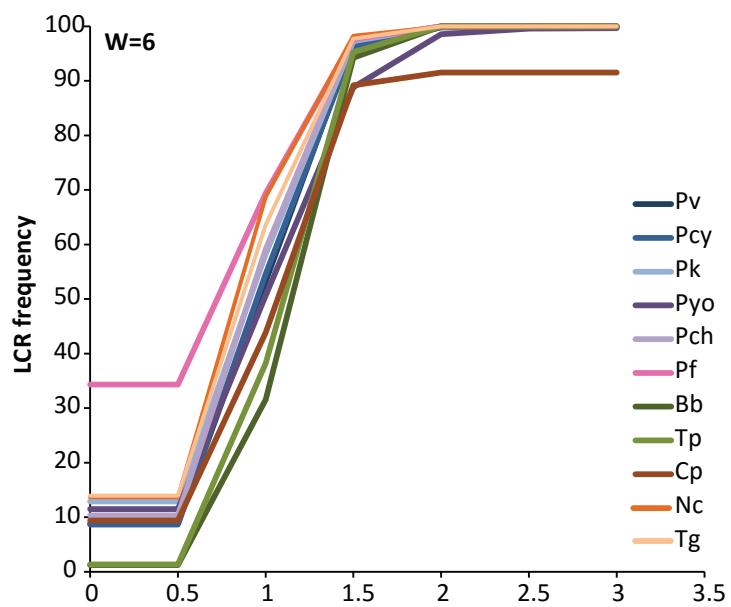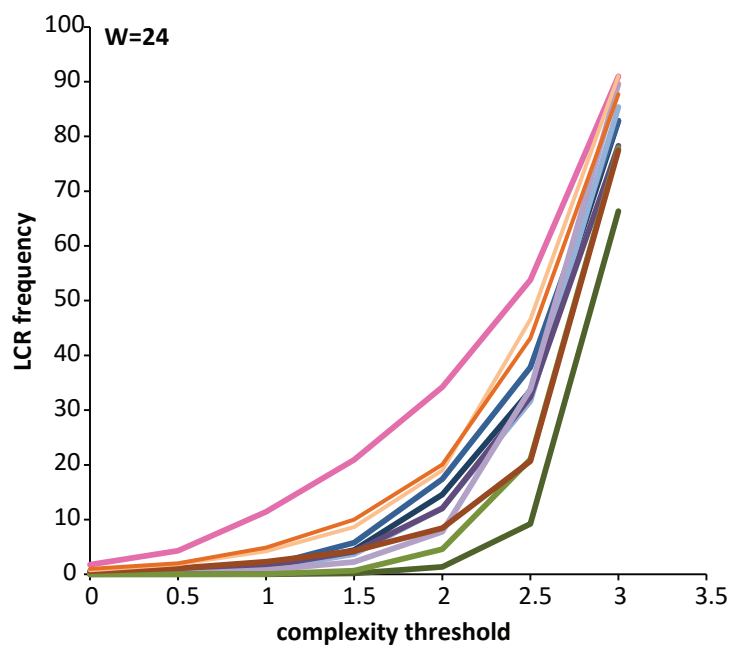

Supplement: Additional file 2: — Profiles of LCRs with window sizes (W) of 6 and 24. Abbreviations are provided in the main text. (PDF 314 kb) [file 12862_2016_625_MOESM2_ESM.pdf]

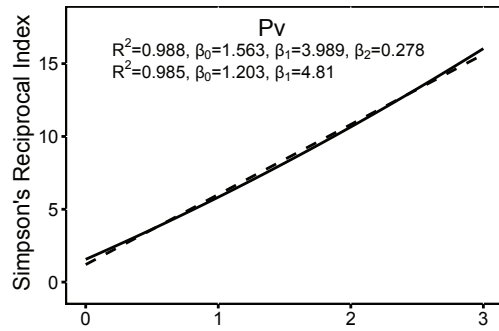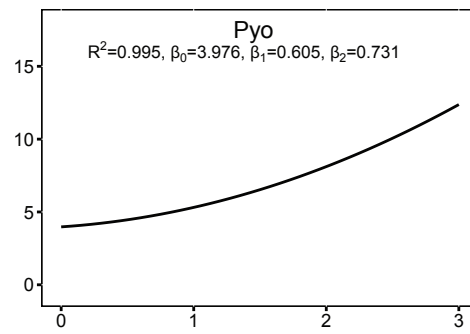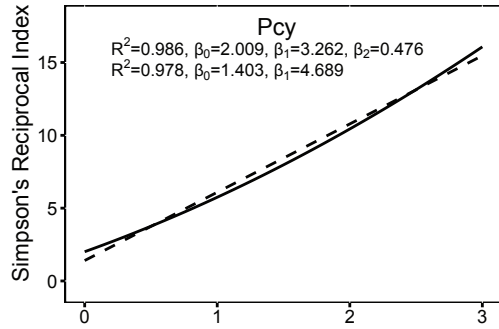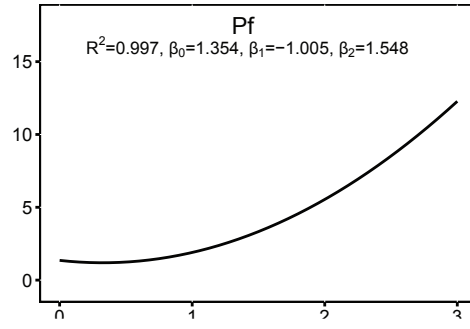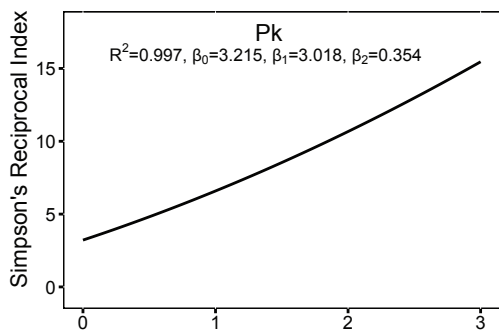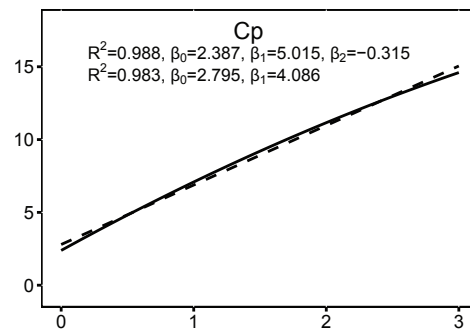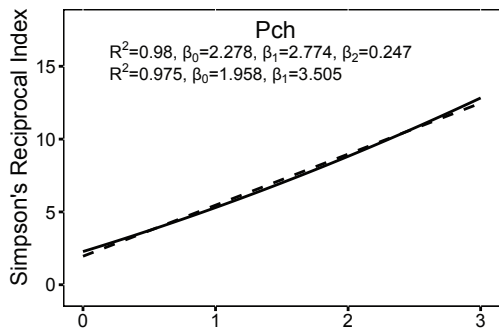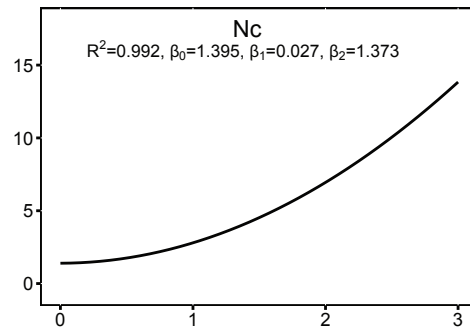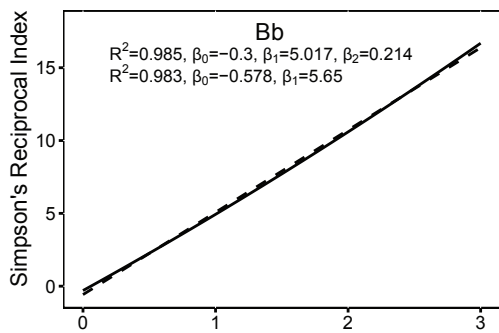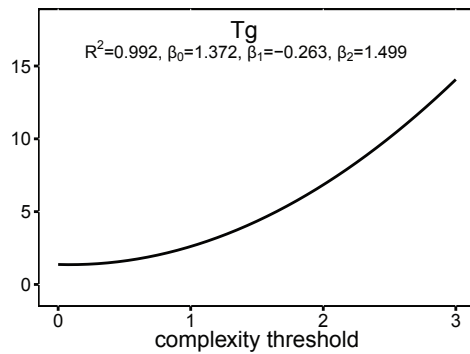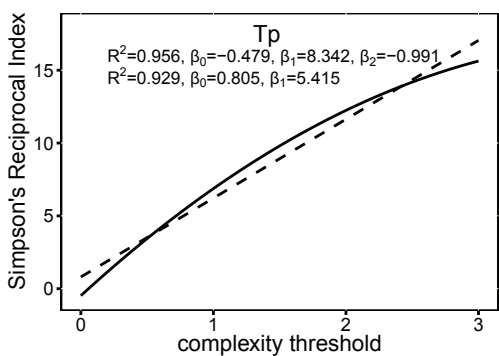

Supplement: Additional file 3: — Species-specific best fit trendlines between complexity thresholds and the Simpson’s Reciprocal Index. Linear and quadratic best fit were tested with a bootstrap method (2000 replicates). Kendall, Spearman, and Pearsons’s coefficients show strong dependency of the two variables. Linear best fits are not shown for those cases where the quadratic best fit is not significantly different from 0. Abbreviations of species names are provided in the main text. (PDF 572 kb) [file 12862_2016_625_MOESM3_ESM.pdf]
